# Repeated Humanin Treatment Attenuates Oxidative Stress, Inflammation, and Apoptosis in Diabetic Cardiac Tissue

**DOI:** 10.3390/biology15131060

**Published:** 2026-07-03

**Authors:** Ferah Bulut, Muhammed Adam, Munevver Gizem Hekim, Mete Ozcan

**Affiliations:** 1 Department of Biophysics, Faculty of Medicine, Firat University, Elazig TR23119, Turkey; ferahbulut94@gmail.com (F.B.); muhamedadamm95@gmail.com (M.A.); 2 Department of Physiology, Faculty of Medicine, Firat University, Elazig TR23119, Turkey; gizemersoz94@gmail.com

**Keywords:** diabetic cardiomyopathy, humanin, oxidative stress, inflammatory cytokines, apoptosis

## Abstract

Diabetes mellitus (DM) markedly increases the risk of cardiovascular complications through mechanisms involving hyperglycemia-induced oxidative stress, inflammation, and apoptosis. Humanin (HN), a mitochondria-derived peptide with established cytoprotective properties, has been reported to exert antioxidant and anti-apoptotic effects in several experimental models. However, its role in diabetic cardiac injury remains insufficiently understood. The present study investigated the protective effects of repeated HN treatment against diabetes-induced cardiac injury in a streptozotocin (STZ)-induced mouse model. Mice were divided into four groups: control, HN-treated, STZ-induced diabetic, and STZ + HN-treated groups (*n* = 10/group). HN (4 mg/kg) was administered daily for 15 consecutive days. Biochemical analyses were performed to evaluate oxidative stress, inflammatory cytokines, and apoptotic markers. STZ-induced diabetes significantly increased oxidative stress markers, pro-inflammatory cytokines, and apoptotic activity while reducing antioxidant defenses and anti-inflammatory cytokines compared with controls. Repeated HN treatment markedly attenuated these alterations and restored redox and inflammatory balance in diabetic cardiac tissue. These findings demonstrate that repeated HN treatment attenuates oxidative stress, inflammation, and apoptosis in the hearts of diabetic mice. The results further suggest that HN may represent a promising therapeutic candidate for limiting diabetes-associated cardiac complications.

## Simple Summary

Diabetes can damage the heart by increasing oxidative stress, inflammation, and cell death, ultimately contributing to the development of cardiovascular complications. Humanin is a naturally occurring peptide produced in mitochondria that has been reported to protect cells from various forms of stress and injury. In this study, we investigated whether repeated Humanin treatment could protect the heart in a mouse model of diabetes. We found that diabetes increased oxidative damage, inflammatory responses, and apoptotic activity in cardiac tissue, whereas Humanin treatment significantly reduced these harmful alterations. These findings suggest that Humanin may help preserve cardiac health under diabetic conditions and could represent a promising therapeutic approach for preventing diabetes-associated heart damage.

## 1. Introduction

Diabetes mellitus (DM) is a major metabolic disorder associated with a substantial increase in cardiovascular morbidity and mortality worldwide [1]. Persistent hyperglycemia contributes to the development of diabetic cardiovascular complications through multiple interconnected mechanisms, including oxidative stress, inflammation, mitochondrial dysfunction, and apoptosis [2]. Among these, oxidative stress is regarded as a central driver of diabetes-associated cardiovascular injury. Chronic hyperglycemia promotes excessive reactive oxygen species (ROS) production, impairs endogenous antioxidant systems, and disrupts mitochondrial homeostasis, thereby contributing to endothelial dysfunction and myocardial damage [2]. Increasing evidence suggests that oxidative stress not only accelerates structural remodeling in diabetic hearts but also amplifies inflammatory signaling pathways involved in disease progression.

Humanin (HN), a 24-amino acid mitochondria-derived peptide, has emerged as an important endogenous mediator involved in cellular adaptation to metabolic and oxidative stress [3]. Initially identified for its cytoprotective properties, HN has subsequently been implicated in the regulation of mitochondrial homeostasis, maintenance of cellular viability, and modulation of stress-responsive signaling pathways [3,4]. Experimental studies indicate that HN enhances insulin sensitivity, preserves β-cell function, and attenuates oxidative stress-associated cellular injury under metabolic stress conditions [3,4]. Mechanistically, HN inhibits mitochondrial Bax translocation, suppresses caspase-dependent apoptosis, and activates pro-survival signaling pathways including AKT, ERK1/2, and STAT3 through the gp130/WSX-1 receptor complex, thereby intersecting with molecular networks governing energy metabolism, inflammation, and cellular survival [5]. Through these coordinated actions, HN is increasingly viewed not only as a cytoprotective peptide but also as a broader regulator of mitochondrial integrity and metabolic homeostasis.

Beyond its intracellular functions, HN also appears to participate in vascular and cardiovascular regulation. Expression of HN within endothelial layers of human arteries and veins suggests a physiological contribution to vascular homeostasis [6,7]. Supporting this concept, exogenous HN markedly attenuates oxidized LDL-induced ROS generation and apoptosis in human aortic endothelial cells, indicating direct protection against oxidative vascular injury [8,9]. Clinical observations further strengthen this association, demonstrating reduced circulating HN concentrations together with increased lipid peroxidation in patients with angina and myocardial infarction, where diminished HN levels independently predict adverse cardiovascular outcomes [8,9]. These cardiovascular observations suggest that HN may exert protective effects extending beyond redox regulation and raise the possibility that HN also modulates inflammatory and injury-related responses within cardiac tissue.

Inflammatory activation represents another key mechanism involved in diabetic cardiac remodeling and functional decline. Sustained hyperglycemia promotes inflammatory activation within the myocardium through oxidative stress-dependent pathways, resulting in excessive cytokine production, immune cell recruitment, and progressive myocardial remodeling [10]. This inflammatory environment contributes not only to structural myocardial injury but also to deterioration of cardiac function during diabetes [11]. In parallel with its antioxidant actions, accumulating evidence suggests that HN exerts important anti-inflammatory effects within the cardiovascular system. In diabetic models, the HN analog S14G-humanin (HNG) reduced circulating and cardiac inflammatory mediators, particularly TNF-α and IL-6, while simultaneously improving cardiac performance and attenuating hypertrophic remodeling [12]. Similarly, HNG suppressed inflammatory cell infiltration and preserved myocardial architecture in pressure-overload and adrenergic heart failure models [13]. Protective effects of HN have also been reported in experimental myocardial infarction, where pretreatment reduced biochemical markers of cardiac injury together with histopathological evidence of inflammatory damage [14].

Mitochondrial dysfunction and excessive ROS production promote activation of intrinsic apoptotic pathways, leading to mitochondrial membrane destabilization, cytochrome c release, and downstream caspase activation within cardiomyocytes [15,16]. In this context, HN has attracted considerable attention due to its direct interactions with mitochondrial cell-death machinery. Experimental evidence demonstrates that HN and its analogs bind pro-apoptotic BAX family proteins, including BAX, Bid, and Bim, thereby preventing BAX activation and mitochondrial translocation [5,17]. Moreover, HN increases the Bcl-2/Bax ratio and inhibits mitochondrial outer membrane permeabilization, ultimately limiting caspase-dependent apoptotic signaling [5,17]. These mechanistic observations support the concept that HN may limit mitochondrial apoptosis and contribute to the preservation of cardiomyocyte survival under diabetic conditions.

Despite growing evidence supporting these protective properties, relatively limited information is available regarding the sustained effects of repeated HN administration on oxidative stress, inflammatory responses, and apoptotic activity in diabetic cardiac tissue [12,13,14]. Therefore, the present study investigated whether repeated HN administration could attenuate diabetes-induced alterations in oxidative stress, inflammatory cytokines, and apoptotic activity in the hearts of STZ-induced diabetic mice and provide further insight into the cardioprotective potential of this mitochondria-derived peptide.

## 2. Materials and Methods

### 2.1. Animals

Forty adult male Balb/C mice (6–8 weeks old, 30 ± 5 g) were obtained from the Experimental Research Center of Fırat University (Elazig, Türkiye). Animals were housed under standard laboratory conditions (23 ± 2 °C, 60 ± 5% humidity, 12 h light/dark cycle) with free access to food and water. After a 7-day acclimatization period, mice were randomly assigned into four groups (*n* = 10/group): Control, HN, streptozotocin-induced diabetic (STZ), and STZ + HN groups.

HN was administered intraperitoneally (i.p.) at a dose of 4 mg/kg once daily for 15 consecutive days. Control and STZ groups received equivalent volumes of phosphate-buffered saline (PBS) according to the same administration schedule used for Humanin treatment. All injections were performed between 09:00 and 10:00. Experimental procedures were approved by the Fırat University Animal Experiments Local Ethics Committee and conducted in accordance with the Guide for the Care and Use of Laboratory Animals (Approval Date: 9 October 2019; Protocol No: 2019/47; Decision No: 2019/19).

### 2.2. Induction of Experimental Diabetes

Experimental diabetes was induced by a single i.p. injection of streptozotocin (STZ; 150 mg/kg; Sigma-Aldrich, St. Louis, MO, USA) dissolved in 0.1 M sodium citrate buffer (pH 4.5) following 12 h fasting. Blood glucose levels were measured from tail vein blood samples 72 h after STZ administration using a glucometer. Diabetes was confirmed by measuring blood glucose levels after STZ administration, and only hyperglycemic animals were included in the study. Mice with blood glucose levels exceeding 250 mg/dL were considered diabetic and included in the study.

### 2.3. Humanin Administration

HN (Catalog No: AS-60886, AnaSpec Inc., Fremont, CA, USA) was dissolved in phosphate-buffered saline (PBS) immediately before administration. HN treatment was initiated three weeks after STZ injection and continued daily for 15 consecutive days via intraperitoneal injection.

At the end of the experimental period, mice were euthanized under anesthesia, and heart tissues were rapidly excised. Left ventricular tissues were collected and stored at −80 °C until biochemical analysis.

### 2.4. Tissue Homogenization

Upon reception, heart tissues were individually placed in labeled Eppendorf tubes. A cold PBS solution was prepared by dissolving 1 tablet in 100 mL. Using a precision scale, the heart tissues were weighed, and a volume of PBS equivalent to nine times the tissue weight was added to the Eppendorf tubes to ensure a consistent PBS-to-tissue ratio based on weight. The left ventricular tissues were fragmented and homogenized using a homogenizer to ensure thorough mixing. Subsequently, the homogenized samples underwent centrifugation at 5000× *g* for 10 min at 4 °C. The resulting samples were then subjected to analysis using the ELISA method.

### 2.5. ELISA

**Figure 1:**
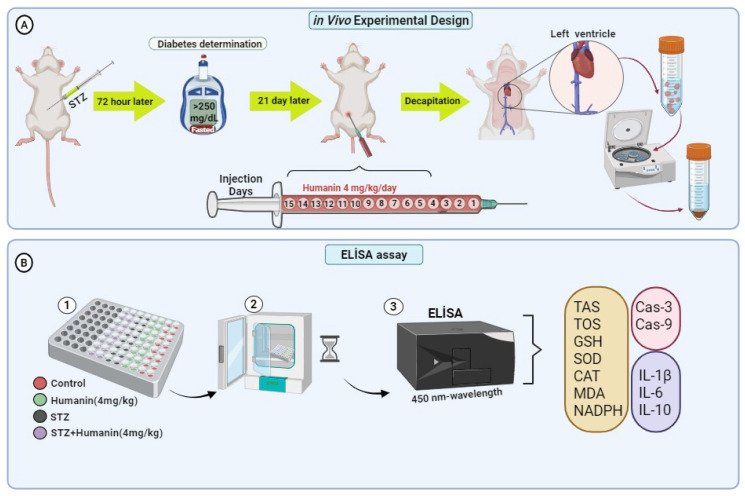
Schematic representation of the experimental design. Streptozotocin (STZ)-induced diabetes was established in mice, followed by repeated Humanin (HN) treatment (4 mg/kg, i.p.) for 15 consecutive days. Left ventricular tissues were collected for evaluation of oxidative stress, inflammatory cytokines, and apoptotic markers. (**A**) In vivo experimental design; (**B**) ELISA assay.

ELISA kits were employed to assess the levels of various biomarkers including total antioxidant status (TAS), total oxidative status (TOS), glutathione (GSH), catalase (CAT), nicotinamide adenine dinucleotide phosphate (NADPH), malondialdehyde (MDA), superoxide dismutase (SOD), caspase-3, caspase-9, interleukin (IL)-1β, IL-6, and IL-10 in mouse left ventricle tissue homogenates. The assay procedure followed the manufacturer’s protocol with the preparation of standard samples and samples at varying concentrations. One well was designated as blank. Specifically, 100 µL of each standard and sample (for caspase-3, caspase-9, IL-1β, IL-6, and IL-10) were added to respective wells of the microtiter plate along with biotin-conjugated antibodies. The enzyme-substrate reaction was terminated by adding a stop solution, and optical signals were recorded using a spectrometer (Multiskan MF, Thermo Fisher Scientific, Vantaa, Finland) at a wavelength of 450 ± 10 nm. The concentrations of TAS, TOS, GSH, CAT, NADPH, MDA, SOD, caspase-3, caspase-9, IL-1B, IL-6 and IL-10 in tissue homogenates were determined by comparing the optical density of the samples with the standard curve provided by the ELISA kits: TAS (E-BC-K801-M, Elabscience, Houston, TX, USA), TOS (E-BC-K802-M, Elabscience, Houston, TX, USA), GSH (E-EL-M0026, Elabscience, Houston, TX, USA), CAT (201-02-1663, SunRed, Shanghai, China), NADPH (201-02-0640, SunRed, Shanghai, China), MDA (E-EL-M0060, Elabscience, Houston, TX, USA), and SOD (E-EL-M2398, Elabscience, Houston, TX, USA), Caspase-3 (E-EL-M0238, Elabscience, Houston, TX, USA), caspase-9 (E-EL-M0241, Elabscience, Houston, TX, USA), IL-1B (E-EL-M0037, Elabscience, Houston, TX, USA), IL-6 (E-EL-M0044, Elabscience, Houston, TX, USA), IL-10 (E-EL-M0046, Elabscience, Houston, TX, USA). Biochemical analyses were performed by investigators blinded to group allocation (Figure 1). The figure was prepared using BioRender.com (https://www.biorender.com; accessed on 26 June 2026).

### 2.6. Statistical Analysis

The results are presented as mean ± standard error of the mean (mean ± SEM). Statistical analysis of the data was conducted using one-way analysis of variance (ANOVA), followed by Tukey’s post hoc test for multiple comparisons. Statistical significance was considered when the *p*-value was less than 0.05 (*p* < 0.05). Effect sizes (Cohen’s d) were calculated to complement statistical significance testing and to estimate the magnitude of group differences.

## 3. Results

### 3.1. Repeated Humanin Treatment Attenuated STZ-Induced Oxidative Stress in the Hearts of Diabetic Mice

The STZ-induced diabetes model caused marked alterations in oxidative stress markers in left ventricular tissue. CAT levels were significantly decreased in diabetic mice compared with the control group (*p* < 0.01–0.0001). Likewise, NADPH content was significantly reduced, whereas MDA levels were significantly increased (*p* < 0.0001). In addition, SOD levels were significantly decreased in diabetic mice compared with the control group (*p* < 0.01–0.0001). These findings indicate impaired antioxidant defense mechanisms, disruption of cellular redox homeostasis, and increased oxidative damage in diabetic cardiac tissue.

**Figure 2:**
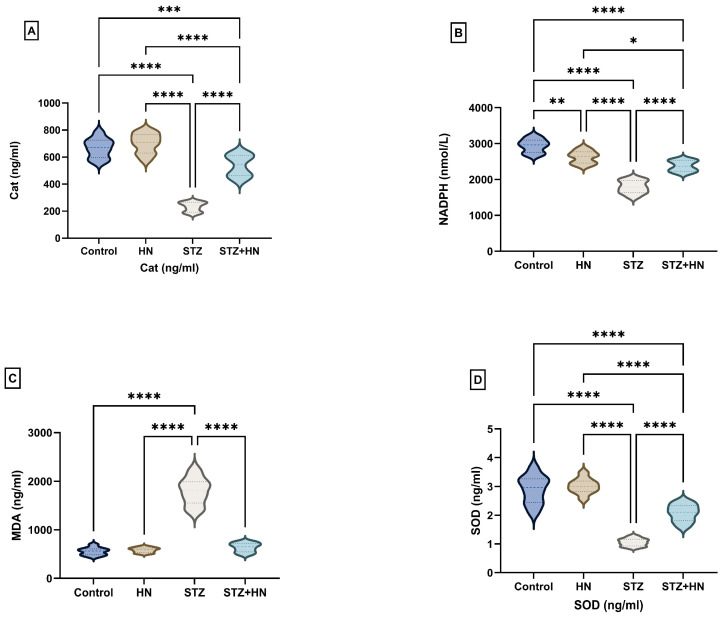
Effects of repeated Humanin (HN) treatment on oxidative stress markers in the hearts of streptozotocin (STZ)-induced diabetic mice. (**A**) CAT level, (**B**) NADPH content, (**C**) MDA level, and (**D**) SOD level in left ventricular tissue. Repeated HN treatment attenuated STZ-induced alterations in oxidative stress markers. Data are presented as violin plots (*n* = 10/group). Statistical analysis was performed using one-way ANOVA followed by Tukey’s post hoc test. * *p* < 0.05, ** *p* < 0.01, *** *p* < 0.001, **** *p* < 0.0001.

Repeated HN administration markedly alleviated these diabetes-induced alterations. CAT levels were significantly increased in the STZ + HN group compared with the STZ group (*p* < 0.01–0.0001). Likewise, NADPH content was restored, whereas MDA levels were significantly reduced (*p* < 0.0001). In addition, SOD levels were significantly increased following HN treatment (*p* < 0.01–0.0001). Oxidative stress parameters in HN-treated diabetic mice were observed to approach the values of the control group. In contrast, administration of HN alone in healthy mice did not result in any significant alteration in oxidative stress markers (Table 1, Figure 2).

### 3.2. Repeated Humanin Treatment Attenuated Apoptotic Activity in the Hearts of Diabetic Mice

**Figure 3:**
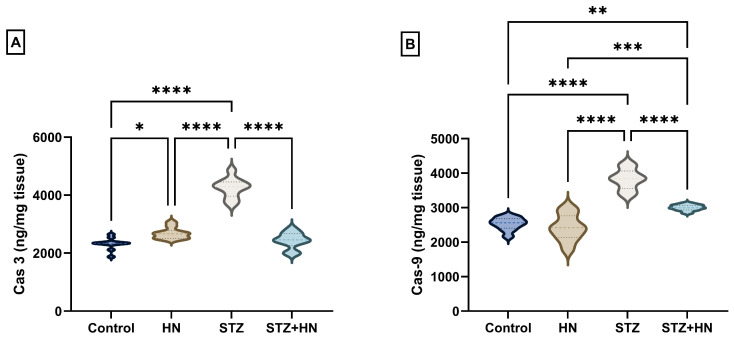
Effects of repeated Humanin (HN) treatment on apoptotic markers in the hearts of streptozotocin (STZ)-induced diabetic mice. (**A**) Caspase-3 and (**B**) caspase-9 levels in left ventricular tissue. Repeated HN treatment attenuated STZ-induced apoptotic activity in diabetic mice. Data are presented as violin plots (*n* = 10/group). Statistical analysis was performed using one-way ANOVA followed by Tukey’s post hoc test. * *p* < 0.05, ** *p* < 0.01, *** *p* < 0.001, **** *p* < 0.0001.

STZ-induced diabetes significantly increased cardiac caspase-3 and caspase-9 levels compared with the control group (*p* < 0.001–0.0001), indicating enhanced apoptotic activity in diabetic cardiac tissue. HN treatment (4 mg/kg) markedly attenuated these alterations by reducing both caspase-3 and caspase-9 levels in diabetic mice relative to untreated diabetic controls (*p* < 0.01–0.0001). In contrast, HN administration alone did not significantly affect cardiac caspase levels in healthy mice (Table 2, Figure 3).

### 3.3. Repeated Humanin Treatment Attenuated Inflammatory Alterations in the Hearts of Diabetic Mice

The STZ-induced diabetes model caused marked alterations in the inflammatory profile of cardiac tissue. Levels of the pro-inflammatory cytokines IL-1β and IL-6 were significantly increased in diabetic mice compared with the control group, whereas a decrease in the anti-inflammatory cytokine IL-10 was observed (*p* < 0.0001). These findings indicate an enhanced inflammatory response in diabetic cardiac tissue.

**Figure 4:**
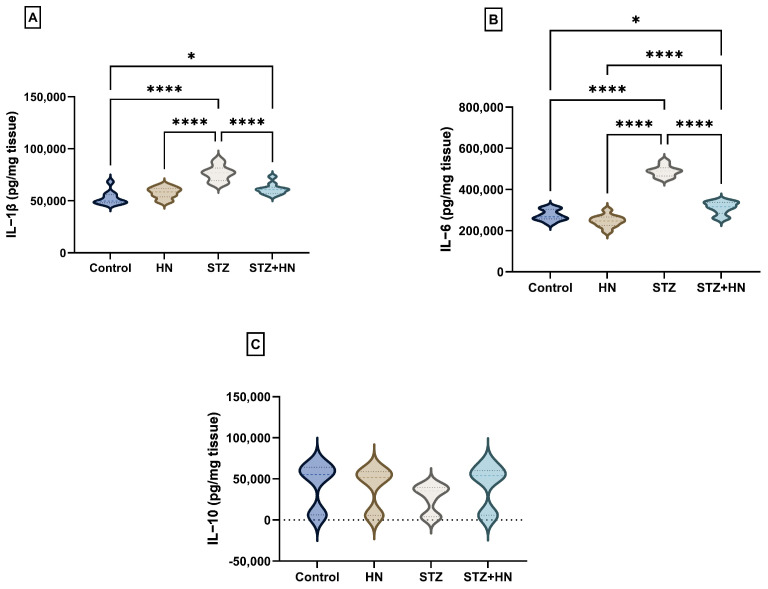
Effects of repeated Humanin (HN) treatment on inflammatory cytokine levels in the hearts of streptozotocin (STZ)-induced diabetic mice. (**A**) IL-1β, (**B**) IL-6, and (**C**) IL-10 levels in left ventricular tissue. Repeated HN treatment attenuated STZ-induced inflammatory alterations in diabetic mice. Data are presented as violin plots (*n* = 10/group). Statistical analysis was performed using one-way ANOVA followed by Tukey’s post hoc test. * *p* < 0.05, **** *p* < 0.0001.

HN administration markedly attenuated diabetes-induced inflammatory alterations. IL-1β and IL-6 levels were significantly lower in the STZ + HN group compared with the STZ group (*p* < 0.001), whereas IL-10 levels showed an increasing trend that did not reach statistical significance. Inflammatory cytokine levels in HN-treated diabetic mice approached those of the control group. In contrast, administration of HN alone in healthy mice did not result in any significant alteration in inflammatory markers (Table 3, Figure 4).

## 4. Discussion

Diabetic cardiac injury is characterized by complex interactions among oxidative stress, inflammation, and mitochondrial apoptosis. The present findings demonstrate that repeated HN administration favorably modulated these interconnected pathways in STZ-induced diabetic mice, supporting a coordinated cardioprotective effect rather than isolated biochemical improvement.

Oxidative stress is considered one of the major mechanisms involved in the development of diabetic cardiac complications [11]. Excessive production of ROS during chronic hyperglycemia impairs cellular antioxidant systems and promotes mitochondrial dysfunction, ultimately contributing to cardiomyocyte injury [12]. In agreement with previous reports, STZ-induced diabetes in the present study reduced cardiac SOD, CAT levels, and NADPH content while increasing MDA levels, indicating severe impairment of redox balance in cardiac tissue [12,13]. Overall, HN treatment restored antioxidant capacity and attenuated lipid peroxidation in diabetic cardiac tissue. These findings are consistent with our previous studies showing that HN exerts potent antioxidant effects under conditions of metabolic and cellular stress [18,19,20]. The protective actions of HN have been associated with suppression of excessive ROS generation and preservation of mitochondrial integrity under stress conditions. Therefore, restoration of redox balance following repeated HN administration may represent an important mechanism underlying its protective effects against diabetes-associated cardiac injury. These findings are also consistent with previous vascular and clinical observations demonstrating reduced HN levels in coronary disease and protective effects against oxidative endothelial injury. In addition, our recent study demonstrated that repeated HN administration restored the circulating levels of several metabolic hormones, including leptin, irisin, ghrelin, and asprosin, in diabetic mice [21]. Together with the present findings, these observations support the concept that HN exerts broad protective actions against diabetes-associated metabolic and tissue-specific abnormalities.

Apoptosis is another critical mechanism contributing to the progression of diabetic cardiomyopathy, particularly in the presence of sustained oxidative stress and mitochondrial dysfunction [22]. Increased ROS production may activate mitochondrial-dependent apoptotic pathways through disruption of mitochondrial membrane stability and activation of downstream caspase signaling cascades. In the current study, diabetic mice exhibited elevated cardiac caspase-3 and caspase-9 levels, reflecting enhanced apoptotic activity in cardiac tissue. Together, the reductions observed in caspase-3 and caspase-9 levels further support the anti-apoptotic properties of HN reported in previous experimental studies [23,24]. Humanin directly interacts with several pro-apoptotic members of the Bcl-2 family, including BAX, Bid, and Bim, thereby preventing mitochondrial membrane destabilization and subsequent caspase activation [17]. In addition, HN promotes cell survival through activation of AKT- and ERK-dependent signaling pathways and preservation of mitochondrial integrity under stress conditions [5]. These physiological actions are particularly relevant in diabetic cardiomyopathy, where chronic oxidative stress and mitochondrial dysfunction contribute to progressive cardiomyocyte loss. Collectively, these observations are consistent with the established role of HN as an endogenous regulator of mitochondrial apoptosis and cellular survival under conditions of metabolic stress.

In addition to oxidative damage and apoptosis, chronic low-grade inflammation is known to play a major role in the progression of diabetes-associated cardiac dysfunction [25]. Hyperglycemia-induced inflammatory activation promotes the release of pro-inflammatory cytokines and disrupts the balance between pro- and anti-inflammatory mediators within cardiac tissue [26]. Consistent with this mechanism, STZ-induced diabetes significantly increased IL-1β and IL-6 levels while reducing IL-10 levels in the hearts of diabetic mice. Collectively, HN treatment shifted the inflammatory profile toward a less pro-inflammatory state by reducing IL-1β and IL-6 levels while restoring IL-10 levels. These findings support previous evidence demonstrating the anti-inflammatory properties of HN in different models of metabolic stress [11,22,23,24]. Although the precise mechanisms remain to be fully clarified, suppression of inflammation together with restoration of redox homeostasis may be closely associated with the protective effects of HN in diabetic cardiac tissue [27].

Beyond its direct antioxidant actions, HN is increasingly recognized as an endogenous modulator of inflammatory responses. Previous studies have demonstrated that HN and its analogs suppress the production of pro-inflammatory cytokines, including TNF-α, IL-1β, and IL-6, while promoting anti-inflammatory signaling under conditions of metabolic and cellular stress [3,28]. The anti-inflammatory effects of HN have been linked to attenuation of oxidative stress-mediated inflammatory activation as well as modulation of intracellular signaling pathways involved in immune regulation. Humanin has also been reported to activate cytoprotective signaling cascades, including AKT and STAT3, which are known to influence inflammatory responses and cellular adaptation to stress [5,17]. Therefore, the reduction in IL-1β and IL-6, together with the restoration of IL-10 observed in the present study, may reflect broader immunomodulatory actions of HN beyond simple suppression of oxidative injury.

The coordinated improvement observed across oxidative, inflammatory, and apoptotic pathways suggests that the protective effects of HN are unlikely to depend on the modulation of a single isolated mechanism. Instead, these findings support the concept that HN may exert integrated cytoprotective actions within diabetic cardiac tissue. Oxidative stress, inflammation, and mitochondrial apoptosis are closely interconnected processes in diabetic cardiomyopathy, where excessive ROS generation amplifies inflammatory signaling and promotes activation of intrinsic apoptotic pathways [29,30,31,32]. Restoration of redox homeostasis may therefore indirectly suppress downstream inflammatory and apoptotic responses, while preservation of mitochondrial integrity may further reinforce cellular resistance to metabolic stress. Given the established interactions of HN with mitochondrial survival pathways and stress-responsive signaling networks [6], the broad biochemical improvement observed in the present study may reflect coordinated regulation of multiple pathological processes rather than isolated biomarker modulation. This integrative response may be particularly relevant in diabetic cardiac injury, where multifactorial mechanisms frequently limit the efficacy of single-target therapeutic strategies.

Recent evidence indicates that mitochondrial dysfunction is a central feature of diabetic cardiomyopathy and involves not only excessive ROS production but also disturbances in mitochondrial dynamics and quality-control mechanisms. Among these regulatory pathways, Mitofusin-2 (Mfn2), a key mediator of mitochondrial fusion and mitochondria–endoplasmic reticulum communication, has emerged as an important determinant of cardiac mitochondrial homeostasis. Dysregulation of Mfn2 under diabetic conditions has been associated with impaired mitochondrial fusion, defective mitophagy, altered calcium handling, increased oxidative stress, and progressive cardiomyocyte dysfunction [33,34]. Experimental studies have further demonstrated that restoration of appropriate Mfn2 activity improves mitochondrial integrity, attenuates ROS accumulation, and reduces apoptotic signaling in diabetic hearts [33,34]. Although Mfn2 and other mitochondrial quality-control pathways were not directly evaluated in the present study, the simultaneous improvement observed in oxidative stress and apoptotic markers following HN treatment is consistent with enhanced preservation of mitochondrial homeostasis. Considering the established role of HN in maintaining mitochondrial integrity and cellular survival under stress conditions, modulation of mitochondrial quality-control mechanisms may represent an additional pathway contributing to its cardioprotective effects in diabetes.

The present study has several limitations that should be considered when interpreting the findings. Histopathological examination and functional cardiac assessments were not performed, limiting direct evaluation of structural preservation and myocardial performance following HN treatment. Likewise, molecular pathways associated with HN-mediated protection, including mitochondrial signaling and downstream survival mechanisms, were not investigated. In addition, gene expression analyses of candidate genes associated with oxidative stress, inflammation, apoptosis, and mitochondrial function were not performed, which may have provided further mechanistic insight into the cardioprotective actions of HN. Assessment of fibrosis-related markers, mitochondrial bioenergetics, and functional cardiac parameters such as echocardiographic measurements would provide a more comprehensive understanding of HN-associated cardioprotection. In addition, the present findings were obtained in an STZ-induced experimental model and should therefore be interpreted within the context of preclinical investigation. Nevertheless, the coordinated improvement observed across oxidative, inflammatory, and apoptotic pathways supports the translational relevance of HN and suggests that mitochondria-derived peptides may represent promising candidates for targeting multifactorial mechanisms involved in diabetic cardiac injury.

## 5. Conclusions

Repeated HN treatment attenuated oxidative stress, inflammation, and apoptotic activity in the hearts of STZ-induced diabetic mice. The restoration of antioxidant defenses together with suppression of inflammatory and mitochondrial apoptotic pathways suggests that HN exerts coordinated cardioprotective effects under diabetic conditions. These findings support the therapeutic relevance of mitochondria-derived peptides such as HN as potential modulators of multifactorial mechanisms involved in diabetic cardiac injury.

## Figures and Tables

**Table 1:** Effects of repeated Humanin administration on oxidative stress and antioxidant defense markers in the left ventricle of diabetic mice.

Group	CAT (ng/mL)	NADPH (nmol/L)	MDA (ng/mL)	SOD (ng/mL)
Control	667.4 ± 22.75 ^cd^	2941 ± 59.37 ^cd^	556.1 ± 24.99 ^c^	2.89 ± 0.15 ^cd^
Humanin (HN)	702.2 ± 23.05 ^cd^	2623 ± 54.90 ^acd^	583.6 ± 16.48 ^c^	3.01 ± 0.04 ^cd^
STZ	232.1 ± 11.23 ^abd^	1793 ± 56.91 ^abd^	1799 ± 87.74 ^abd^	1.06 ± 0.04 ^abd^
STZ + HN	538.2 ± 25.03 ^abc^	2376 ± 47.84 ^abc^	637.9 ± 30.19 ^c^	2.08 ± 0.09 ^abc^

Values are presented as mean ± SE (*n* = 10 animals per group). CAT, catalase; NADPH, nicotinamide adenine dinucleotide phosphate; MDA, malondialdehyde; SOD, superoxide dismutase. ^a^: *p* < 0.05 versus the Control group; ^b^: *p* < 0.05 versus the Humanin (HN) group; ^c^: *p* < 0.05 versus the STZ group; ^d^: *p* < 0.05 versus the STZ + HN group.

**Table 2:** Effects of repeated Humanin administration on apoptotic markers in the left ventricle of diabetic mice.

Group	Caspase-3 (ng/mg Tissue)	Caspase-9 (ng/mg Tissue)
Control	2324 ± 66.57 ^bc^	2531 ± 58.21 ^cd^
Humanin (HN)	2682 ± 60.03 ^cd^	2420 ± 120.7 ^cd^
STZ	4254 ± 107.4 ^abd^	3824 ± 95.07 ^abd^
STZ + HN	2438 ± 86.94 ^c^	3001 ± 26.75 ^abc^

Values are presented as mean ± SE (*n* = 10 animals per group). Caspase-3 and Caspase-9 were determined in left ventricular tissue homogenates. ^a^: *p* < 0.05 versus the Control group; ^b^: *p* < 0.05 versus the Humanin (HN) group; ^c^: *p* < 0.05 versus the STZ group; ^d^: *p* < 0.05 versus the STZ + HN group.

**Table 3:** Effects of repeated Humanin administration on inflammatory cytokine levels in the left ventricle of diabetic mice.

Group	IL-1β (pg/mg Tissue)	IL-6 (pg/mg Tissue)	IL-10 (pg/mg Tissue)
Control	52,524 ± 2073 ^cd^	274,892 ± 8477 ^cd^	43,981 ± 8434 ^cd^
Humanin (HN)	57,734 ± 1691 ^c^	246,959 ± 9088 ^cd^	40,327 ± 7725 ^cd^
STZ	76,426 ± 2381 ^abd^	489,227 ± 8586 ^abd^	26,793 ± 6294 ^ab^
STZ + HN	61,236 ± 1671 ^ac^	311,286 ± 9802 ^abc^	32,264 ± 8189 ^ab^

Values are presented as mean ± SE (*n* = 10 animals per group). IL, interleukin. ^a^: *p* < 0.05 versus the Control group; ^b^: *p* < 0.05 versus the Humanin (HN) group; ^c^: *p* < 0.05 versus the STZ group; ^d^: *p* < 0.05 versus the STZ + HN group.

## Data Availability

The data presented in this study are available from the corresponding author upon reasonable request.
